# Supplementary material for: Bayesian network models identify co-operative GPCR:G protein interactions that contribute to G protein coupling
Source: bioRxiv. 2023 Oct 12:2023.10.09.561618. Preprint. [Version 1] doi: 10.1101/2023.10.09.561618 (PMC10592737; doi:10.1101/2023.10.09.561618)
Supplement: Supplement 9 [file NIHPP2023.10.09.561618v1-supplement-9.pdf]

bioRxiv preprint doi: https://doi.org/10.1101/2023.10.09.561618; this version posted October 12, 2023. The copyright holder for this preprint (which was not certified by peer review) is the author/funder, who has granted bioRxiv a license to display the preprint in perpetuity. It is made available under aCC-BY 4.0 International license.

Supplemental Figure Legends

Supplemental Figure 1. a. Root-mean-square deviation (RMSD) values for TM backbone atoms in the transmembrane helices.

Supplemental Figure 2. a. Table demonstrating the total number of ICL3 residues across GPCRs analyzed in this study and the number of resolved residues in the three-dimensional structures used for the MD simulations. **b.** Table demonstrating the total number of TM5 interactions with any G protein region and the number of contacts that TM5 makes only with hgh4-loop of G protein core region.

Supplemental Figure 3. Evaluation of Bayesian Network Analysis Robustness. a. Cumulative Distribution Function of all network scores with various topology perturbations. Red dashed line indicates the placement of score of the original networks. **b.** Cumulative Distribution Function of all network scores with data resampling. Red dashed line indicates the placement of score of the original networks.

Supplemental Figure 4. Alignment of Go and Gq protein sequences. a. BN-predicted cooperativity hotspots are marked in colored font according to the subtype: green – Go/Gi protein, blue – Gq protein. Selectivity hotspots are shown in red boxes.

Supplemental Figure 1

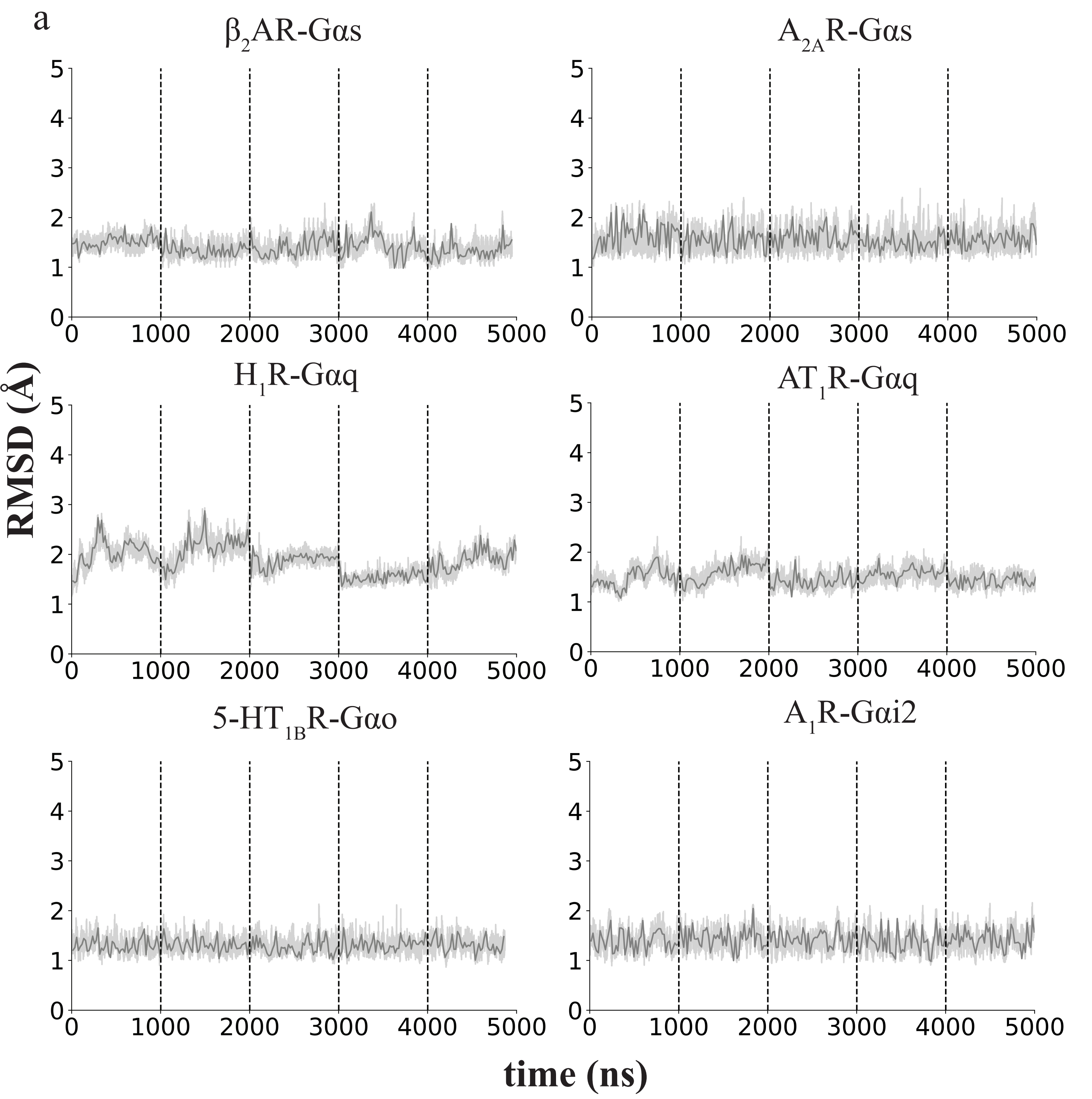

Supplemental Figure 2

a

Presence of ICL3 residues in 3D structures

# of ICL3 residues	5-$HT_{1B}R$	$β_2AR$	H_1R	AT1R	A1R	A2AR
present in receptor	59	25	176	8	6	6
resolved in 3D structure	0	2	0	8	0	0

b

Contribution of hgh4-loop contacts with TM5 to total number of TM5 contacts

# of TM5 contacts	5-$HT_{1B}R$	$β_2AR$	H_1R	AT1R	A1R	A2AR
with all G protein regions	53	156	51	12	73	93
with hgh4-loop	0	28	0	0	0	29

Supplemental Figure 3

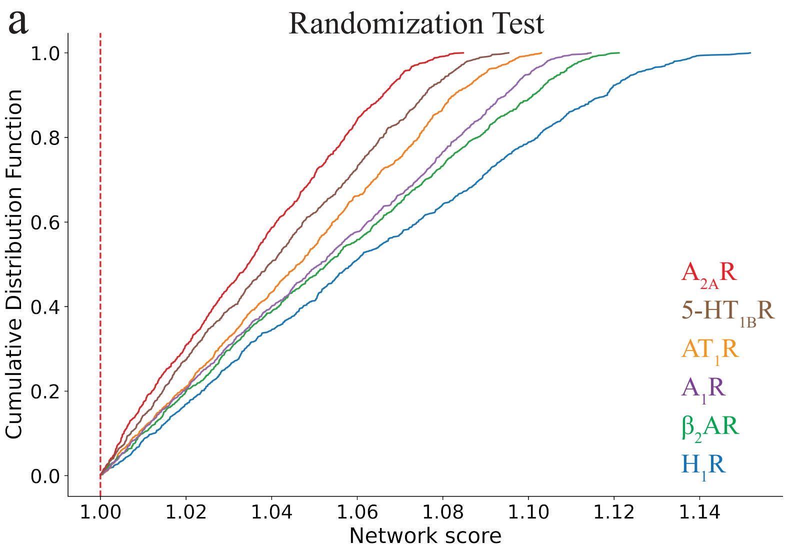

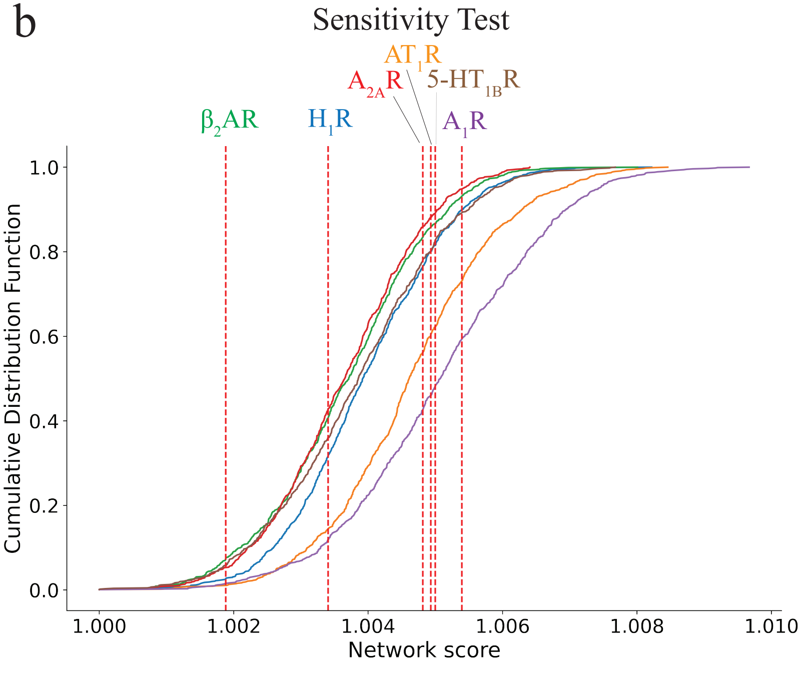

Supplemental Figure 4

a

```
                HN                                    S1           H1
gnao_human      ------MGCTLSAEERAALERSKAIEKNLKEDGISAAKDVKLLLLGAGESGKSTIVKQMK 54
gnaq_human      MTLESIMACCLSEEAKEARRINDEIERQLRRDKRDARRELKLLLLGTGESGKSTFIKQMR 60
                      *.* ** * : * . .. **::*:.*  .* :::******:*******::***:

                   AHD domain
gnao_human      IIHEDGFSGEDVKQYKPVVYSNTIQSLAAIVRAMDTLGIEYGDKERKADAKMVCDVVSRM 114
gnaq_human      IIHGSGYSDEDKRGFTKLVYQNIFTAMQAMIRAMDTLKIPYKYEHNKAHAQLVREVDVEK 120
                *** .*:*.** : :. :**.* : :: *::****** * *  :..**.*::* :*  .

gnao_human      EDTEPFSAELLSAMMRLWGDSGIQECFNRSREYQLNDSAKYYLDSLDRIGAADYQPTEQD 174
gnaq_human      VS--AFENPYVDAIKSLWNDPGIQECYDRRREYQLSDSTKYYLNDLDRVADPAYLPTQQD 178
                 .   *.   :.*:  **.* *****::* *****.**:****:.***:.   * **:**
                          S2        S3         H2             S4
gnao_human      ILRTRVKTTGIVETHFTFKNLHFRLFDVGGQRSERKKWIHCFEDVTAIIFCVALSGYDQV 234
gnaq_human      VLRVRVPTTGIIEYPFDLQSVIFRMVDVGGQRSERRKWIHCFENVTSIMFLVALSEYDQV 238

                        H3                    S5      HG
gnao_human      LHEDETTNRMHESLMLFDSICNNKFFIDTSIILFLNKKDLFGEKIKKSPLTICFPEYTGP 294
gnaq_human      LVESDNENRMEESKALFRTIITYPWFQNSSVILFLNKKDLLEEKIMYSHLVDYFPEYDGP 298
                * *.:. ***.**  ** :* .  :* ::*:*********: ***  * *.  **** **
                H4               h4s6      S6         H5
gnao_human      NTYEDAA-AYIQAQFESKNRSPNKE--IYCHMTCATDTNNIQVVFDAVTDIIIANNLRGC 351
gnaq_human      QRDAQAAREFILKMFVDL--NPDSDKIIYSHFTCATDTENIRFVFAAVKDTILQLNLKEY 356
                :   :**  :*   * .   * . :* **.*:******:**:.** **.* *:  **:

gnao_human      GLY         354
gnaq_human      NLV         359
                .*
```
